# Orthodontic finishing errors detected in board-approved cases: common types and prediction

**DOI:** 10.1590/2177-6709.29.4.e2424102.oar

**Published:** 2024-09-02

**Authors:** José VALLADARES-NETO, Lincoln Issamu NOJIMA, Heloísio de Rezende LEITE, Matheus Melo PITHON, Adilson Luiz RAMOS, Luís Antônio de Arruda AIDAR, Roberto ROCHA, Carlos Alberto Estavanell TAVARES, Jonas CAPELLI-JR, Roberto Carlos Bodart BRANDÃO, Marcos Alan Vieira BITTENCOURT, Guilherme de Araújo ALMEIDA, Mirian Aiko Nakane MATSUMOTO

**Affiliations:** 1 Brazilian Board of Orthodontics and Dentofacial Orthopedics (Brazil).; 2 Federal University of Goiás, School of Dentistry, Department of Orthodontics (Goiânia/GO, Brazil).; 3 Federal University of Rio de Janeiro, School of Dentistry, Department of Orthodontics (Rio de Janeiro/RJ, Brazil).; 4 Pontifical Catholic University of Minas Gerais, School of Dentistry, Department of Orthodontics (Belo Horizonte/MG, Brazil).; 5 State University of Southwest Bahia, School of Dentistry, Department of Orthodontics and Pediatric Dentistry (Vitória da Conquista/BA, Brazil).; 6 State University of Maringá, School of Dentistry, Department of Orthodontics (Maringá/PR, Brazil).; 7 Santa Cecilia University, School of Dentistry, Department of Orthodontics (Santos/SP, Brazil).; 8 Federal University of Santa Catarina, School of Dentistry, Department of Stomatology (Florianópolis/SC, Brazil).; 9 Private clinic (Porto Alegre/RS, Brazil).; 10 State University of Rio de Janeiro, School of Dentistry, Department of Orthodontics (Rio de Janeiro/RJ, Brazil).; 11 Federal University of Espírito Santo, School of Dentistry, Department of Orthodontics (Vitória/ES, Brazil).; 12 Federal University of Bahia, School of Dentistry, Department of Orthodontics (Salvador/BA, Brazil).; 13 Federal University of Uberlândia, School of Dentistry, Department of Orthodontics (Uberlândia/MG, Brazil).; 14 University of São Paulo, School of Dentistry, Department of Pediatric Dentistry (Ribeirão Preto/SP, Brazil).

**Keywords:** Dental finishing, Treatment outcome, Orthodontics, Finalização ortodôntica, Resultado de tratamento, Ortodontia

## Abstract

**Objective:**

To report and rank orthodontic finishing errors recorded in the clinical phase of the Brazilian Board of Orthodontics and Dentofacial Orthopedics (BBO) examination and correlate pretreatment case complexity with orthodontic treatment outcomes.

**Materials and Methods:**

This single-center cross-sectional survey collected retrospective data from the clinical phase of BBO examinations between 2016 and 2023. The quality of orthodontic clinical outcomes of each case was assessed by means of the Cast-Radiograph Evaluation (CRE), while case complexity was evaluated using the Discrepancy Index (DI), both tools provided by the American Board of Orthodontics. Survey items were analyzed using descriptive statistics, and a correlation analysis between total CRE and DI scores (*p*<0.05) was also performed.

**Results:**

A total of 447 orthodontic records was included. Orthodontic finishing errors were often observed, and no case was completely perfect. In the total CRE score, an average of 15 points was discounted for each case. Most frequently found issues involved problems with alignment, buccolingual inclination, marginal ridge, and occlusal relationship. The median DI score for initial case complexity was 22.0 (range 10.0 - 67.0). There was no significant correlation between the DI and CRE scores (*p*=0.106).

**Conclusion:**

Orthodontic finishing errors are inevitable, even in well-finished board-approved cases. Rotation, excessive buccolingual inclination, and discrepancies in marginal ridges are the most frequently observed areas of concern, in that order. Moreover, while case complexity, determined by DI, can impact orthodontic planning and pose challenges for clinicians, the study did not consider it a determining factor in predicting treatment outcomes.

## RESUMO

**Objetivo:**

Identificar as principais falhas de finalização ortodôntica registradas pelo Board Brasileiro de Ortodontia e Ortopedia Facial (BBO) e correlacionar a severidade inicial do caso com a qualidade da finalização ortodôntica.

**Material e Métodos:**

Esse estudo transversal unicêntrico coletou dados retrospectivos obtidos durante a Fase II dos exames do BBO, entre 2016 e 2023. A qualidade da finalização ortodôntica foi avaliada pelo Sistema Objetivo de Avaliação (SOA), e a severidade inicial do caso, pelo Índice do Grau de Complexidade (IGC), ambas ferramentas do *American Board of Orthodontics*. Os dados foram analisados por meio de estatística descritiva, e a análise de correlação entre os escores do SOA e IGC foi calculada (*p* < 0,05).

**Resultados:**

447 casos ortodônticos foram incluídos. Falhas de finalização ortodôntica foram detectadas em todos os casos. Em média, 15 pontos foram descontados no escore final do SOA. As falhas mais comuns envolveram problemas de alinhamento, inclinação vestibulolingual, e nivelamento das cristas marginais. O escore médio do IGC foi de 22,0 (amplitude: 10,0 - 67,0). Não houve correlação significativa entre os escores de IGC e SOA (*p* = 0,106).

**Conclusão:**

As falhas de finalização ortodôntica são inevitáveis, mesmo em casos aprovados pelo BBO. Rotação, inclinação vestibulolingual excessiva e desníveis nas cristas marginais, nessa ordem, são as áreas que merecem mais atenção. Além disso, a severidade inicial do caso, determinada pelo IGC, não foi considerada um fator determinante na predição da qualidade final do tratamento, embora ela possa impactar o planejamento e representar desafios para os clínicos.

## INTRODUCTION

Perfect finishing of dental occlusion is a critical aspect of orthodontic practice, and often involves errors. Orthodontic finishing errors typically include mistakes or inaccuracies occurring during the treatment process, due to factors such as incorrect diagnosis, inadequate treatment objectives and planning, or technical misconception. Such errors can lead to suboptimal results, and should be identified and minimized.^1^ ^,^ ^2^

In 1998, the American Board of Orthodontics (ABO) committee introduced the ‘Objective Evaluation System’, which later became known as the ‘Cast-Radiograph Evaluation’ (CRE).^3^ This standardized objective tool is used to assess the quality of orthodontic treatment outcomes, based on posttreatment dental casts and panoramic radiographs. This grading system was implemented to enhance the objectivity, reliability, and validity of clinical examinations, particularly in the third stage of ABO certification. The reporting of finishing errors was initially introduced in the context of the ABO exam in 1999.^1^ ^,^ ^3^ Its applicability, however, has expanded to other purposes, such as assessing long-term posttreatment changes and comparing outcomes of different orthodontic treatment approaches.^4^ ^-^ ^8^ The CRE has also been applied to evaluate the performance of university graduate orthodontics programs and in private clinics.^9^ ^-^ ^11^ In summary, this standardized tool has become essential for orthodontists and researchers, as it provides an objective means of evaluating the quality of orthodontic treatment outcomes.

Traditionally, occlusal finishing was manually assessed using physical resources, such as dental casts and printed models. While virtual methods have also been developed for measuring on digital models, studies indicate that the quantitative assessment of occlusion through digital approaches still lacks reliability, whether based on user-selected landmarks or conducted through automated means.^12^ ^-^ ^14^

The Discrepancy Index (DI) is another objective tool developed by the ABO to assess case complexity before orthodontic treatment.^15^ It is based on measurements taken from pretreatment dental models, and from cephalometric and panoramic radiographs. It acts as a quantitative indicator of the initial severity of malocclusion and the level of difficulty associated with its correction during orthodontic treatment. In Brazil, both CRE and DI criteria have been adopted by the Brazilian Board of Orthodontics and Dentofacial Orthopedics (BBO) to evaluate candidates during certification and recertification exams.^16^

Orthodontic examination boards worldwide, supported by case-based examinations, have made major efforts in identifying these finishing errors. However, the recent introduction of the Scenario-Based Clinical Examination by the ABO marks a notable shift in the evaluation process, by discontinuing case requirements.^17^ Despite this change, the recognition and addressing of orthodontic finishing errors remain essential for improving orthodontic quality and providing valuable educational insights for clinicians, educators, and students to refine skills and implement preventive measures. Furthermore, predicting the occurrence of these errors can be challenging yet essential for optimizing treatment outcomes. However, within the realm of board examinations, there are no evidence-based studies, as certain pre-existing information is solely based on informal accounts.^1^ ^,^ ^3^

In view of such knowledge gaps, this study focuses on Brazilian candidates for the board certification exam. Hence, the primary objective of the study was to report and rank the occurrence of orthodontic finishing errors detected during the clinical phase of the BBO examination. In addition, a secondary objective was to establish a correlation between the complexity of pretreatment cases and the clinical outcomes of orthodontic treatment. The hypothesis being put forward is that orthodontic finishing errors are expected and unlikely to be predicted.

## MATERIAL AND METHODS

This single-center cross-sectional survey was based on secondary databases from files collected during BBO exams. The protocol was approved by the Research Ethics Committee at the Federal University of Goiás (CAAE: 71120123.0.0000.5083) and was in full compliance with Brazilian Resolutions 466/12 and the Declaration of Helsinki. The STROBE guidelines for observational studies were followed.^18^

### DATA COLLECTION

Data were obtained from BBO files at a digital platform (*www.bbo.org.br*) for each case approved during the clinical phase of the BBO examinations, from 2016 to 2023. For each candidate, the certification exam demanded six cases, of which at least three had a minimum DI score of 10 and the others, a minimum of 20.^16^ The clinical cases had been treated exclusively by Brazilian orthodontists, who took the examination on a voluntary basis. The data were retrieved from the BBO files, taking into consideration the CRE (primary outcome) and the DI (secondary outcome). The evaluation process had involved the prior training and calibration of examiners, and simultaneous assessment by pairs of examiners for each case presented. The exclusion criterion for this study was a DI score lower than 10 (minimal severity score) and a CRE higher than or equal to 30 (minimum score for quality finishing).

### CAST-RADIOGRAPH EVALUATION (CRE)

**Figure 1:**
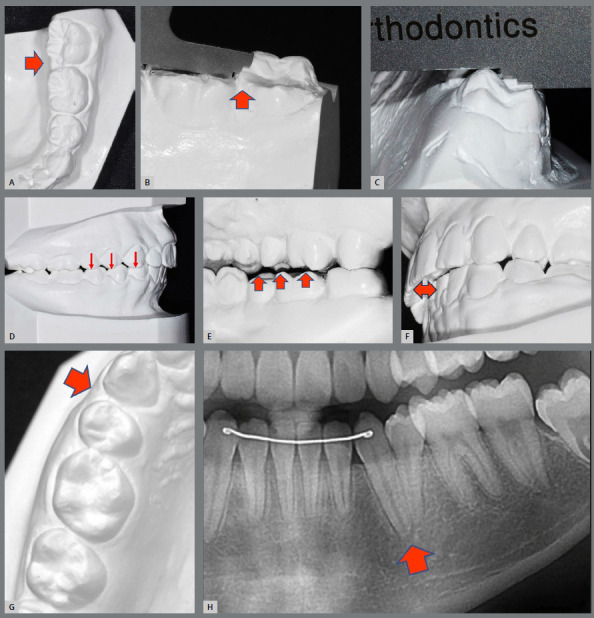
Cast-Radiograph Evaluation (CRE) criteria, which detected errors in the following categories: **A**) alignment, **B**) marginal ridge leveling, **C**) buccolingual inclination, **D**) occlusal relationship, E) occlusal contacts, **F**) overjet, **G**) interproximal contacts, and **H**) root parallelism.

The orthodontic clinical outcomes were evaluated according to the CRE as described by Casko et al.,^3^ which is based on the following grading parameters: 1) tooth alignment, 2) marginal ridge leveling, 3) buccolingual inclination, 4) occlusal relationship, 5) occlusal contacts, 6) overjet, 7) interproximal contacts, and 8) root parallelism (root angulation) (Fig 1). Posttreatment dental casts or printed models were used to analyze and score parameters 1 to 7, while panoramic radiography was used for root parallelism.^19^

**Figure 2:**
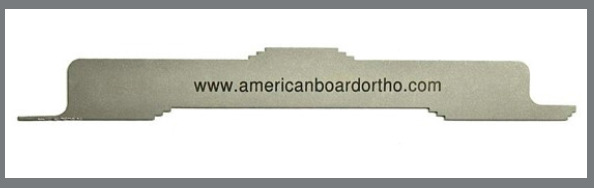
ABO measuring gauge.

The models had been simultaneously graded by two different pre-calibrated examiners using the ABO measuring gauge (Fig 2). Partial scores were assigned to each category evaluated (1 to 8). Points were deducted (1 or 2) for each item according to how far individual teeth deviated from the standards established by the ABO. A zero score indicated an absence of errors, while more negative scores indicated the presence of errors and its corresponding severity (Table 1).^3^ ^,^ ^16^ ^,^ ^19^

**Table 1:** Cast-Radiograph Evaluation (CRE) methodology.

Category	Local and assessment	Measurement or visualization	Point subtracted
Alignment	Anterior and posterior teeth rotation, with different references to assess proper alignment: » Upper anterior teeth: incisal edges and lingual surfaces; » Lower anterior teeth: incisal edges and labial surfaces; » Upper posterior teeth: mesiodistal central groove; » Lower posterior teeth: buccal cusps.	< 0.5 mm 0.5 to 1 mm > 1 mm	0 1 2
Marginal ridge leveling	Posterior teeth, in each interproximal contact, except on the distal ridge of lower first premolar	< 0,5 mm 0,5 to 1 mm > 1 mm	0 1 2
Buccolingual inclination	Posterior teeth, except measurements of lower premolars	< 1 mm 1 to 2 mm > 2 mm	0 1 2
Occlusal relationship	Anteroposterior relationship involving canine to second molar. Upper buccal cusps should occlude within 1 mm of the lower interproximal embrasures.	< 1 mm 1 to 2 mm > 2 mm	0 1 2
Occlusal contacts	Posterior teeth, considering the functioning upper cusps and the correspondent lower functioning cusps and pits.	Contacting Non-contacting < 1 mm Non-contacting > 1 mm	0 1 2
Overjet	Anterior and posterior teeth. Measurements obtained from each upper tooth. Anterior teeth should be in contact. Lower buccal cusp of posterior tooth should occlude with the upper central groove.	Anterior contact: 0 mm Anterior: Non-contacting < 1 mm Anterior: Non-contacting > 1 mm Posterior coordination: 0 mm Posterior: incoordination < 1 mm Posterior incoordination > 1 mm	0 1 2 0 1 2
Interproximal contacts	Effective or spaced interproximal contact evaluated from occlusal perspective.	< 0.5 mm 0.5 to 1 mm > 1 mm	0 1 2
Root parallelism	Evaluated from panoramic radiograph. Disregard significant root dilaceration.	Root parallelism Root non-parallel Roots in contact	0 1 2

In accordance with the ABO scoring system, a lower sum of category score (total CRE) indicated a better finished case, while a higher score showed a less well-finished case.^3^

### DISCREPANCY INDEX (DI)

Case complexity was supported by DI scores and calculated for each case based on the recommended pretreatment exam, or in other words, on the dental models, and cephalometric and panoramic radiographs.^15^ The components of the DI included overjet, overbite, anterior open bite, crowding, occlusion, lingual posterior crossbite, buccal posterior crossbite, and cephalometric values, such as ANB, SN.GoGn and IMPA. The score could vary from 10 points (minimum complexity for exclusion criteria), and the higher the score, the greater the complexity of the case. 

### STATISTICAL ANALYSIS

Given its retrospective design, it should be noted that the board committee consistently achieved high levels of inter-examiner reliability, ranging from good to excellent, prior to the collection of the CRE and DI data. 

Variables were not normally distributed, based on skewness statistics and confirmed by Shapiro-Wilk test (*p*<0.05). Descriptive statistics (median and interquartile ranges 25-75%) were computed for all survey items, and the Spearman correlation analysis was used for statistical evaluation between total CRE and DI scores. The statistical analysis was performed using Jamovi software (version 2.3, Australia, retrieved from *www.jamovi.org*) with a significance level of 5%.

## RESULTS

**Figure 3:**
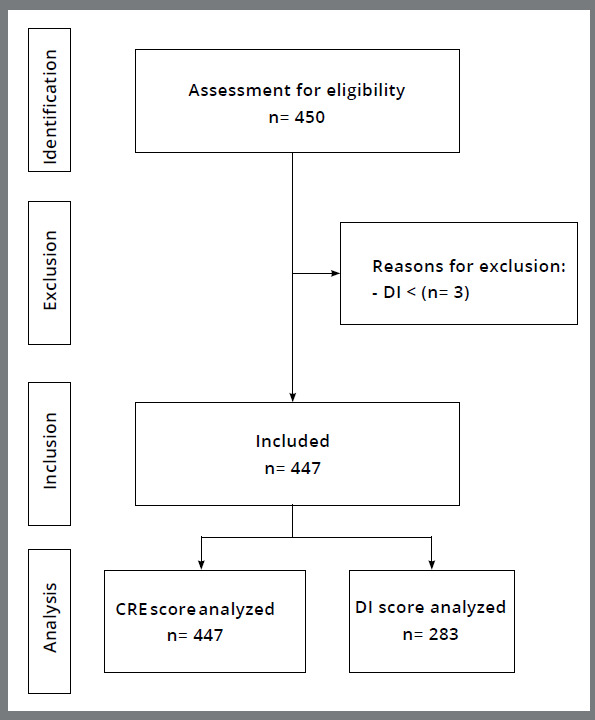
Flow chart illustrating sample selection.

In the last eight years (2016 to 2023), seven annual exams were performed. In 2020, there was no exam, due to the COVID-19 pandemic. Over this period, 75 orthodontists underwent BBO certification, totaling 450 cases. Three cases were excluded, as they presented a DI less than 10, thereby giving a total of 447 cases analyzed (Fig 3). 

**Figure 4:**
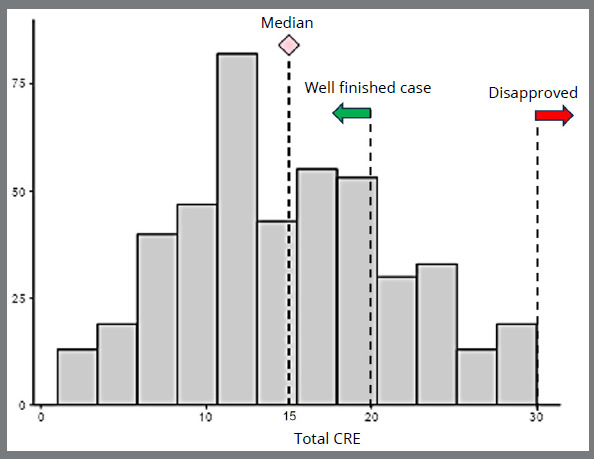
Distribution of total CRE score showing median and cutoff values (n= 447).

**Figure 5:**
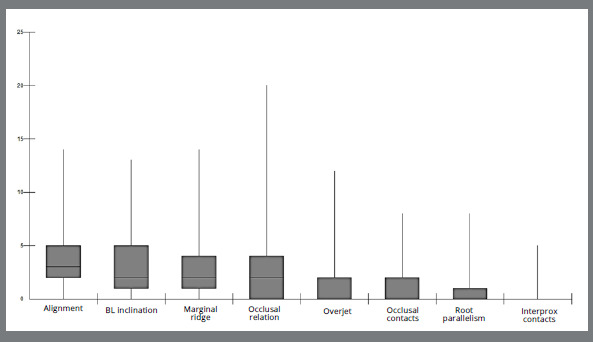
Boxplot of CRE score components (n= 447).

Orthodontic finishing errors were common, and no case yielded a zero score, that is, an absence of errors. The median score for the total CRE was 15 [IQR (25%-75%): 10 - 19; range: 1 to 30) (Fig 4). The highest scored finishing errors involved problems with tooth alignment [median: 3 points/per case, IQR (25%-75%): 2 to 5, range: 0 to 14], buccolingual inclination [median: 2 points/per case, IQR (25%-75%): 1 to 5, range: 0 to 13], marginal ridge level [median: 2.0 points/per case, IQR (25%-75%): 1 to 4, range: 0 to 14], and occlusal relationship [median: 2.0 points/per case, IQR (25%-75%): 0 to 4, range: 0 to 20]. Overjet, occlusal contacts, root parallelism, and interproximal contacts were found to be the least compromised criteria, each with a median score of 0 (Fig 5). 

**Figure 6:**
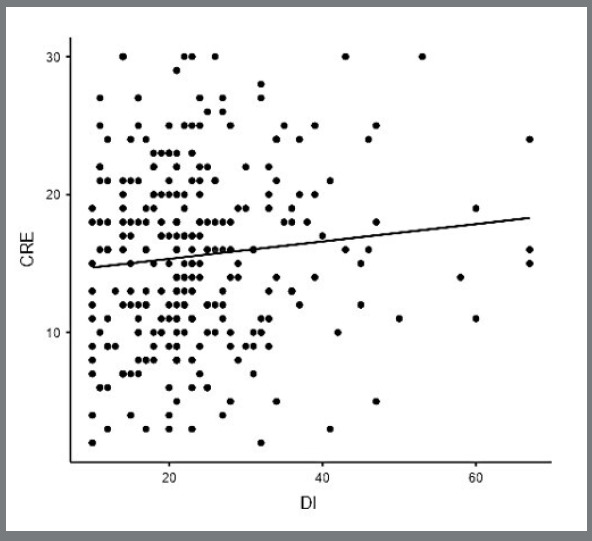
Scatter plot of CRE and corresponding DI score (n= 283).

Only total DI data from BBO exams for the years 2019 to 2023 were available to be computed (Fig. 3). Hence, 283 cases [median: 22.0, IQR (25%-75%): 17.0 to 28.0; range: 10.0 to 67.0] were computed and analyzed according to the corresponding CRE. There was no significant correlation between total CRE and the corresponding DI scores (*p*=0.106) (Fig 6).

## DISCUSSION

The primary mission of the BBO certification exam is to endorse proper orthodontic practice and certify the clinical excellence of treatment outcomes.^16^ The sample included in this study encompassed the years 2016 to 2023 and reflected its standardized methodology for data collection. It should be noted that no previous studies with a large dataset have been published focusing on a case-based board examination. The main findings of this study indicate that orthodontic finishing details are susceptible to errors even in approved cases, thereby highlighting the high sensitivity of the CRE method in detecting subtle alterations. Despite the occurrence of finishing errors, cases achieved a median score of 15 for total CRE, which is typically considered well-finished by the ABO criteria, as scores below 20 are classified in this way. On the other hand, a total CRE score of greater than 30 was an automatic criterion for case rejection (Fig 4).^3^ ^,^ ^19^

This outcome generally indicated better case finalization, compared to other settings examined, such as university graduate programs and private clinics, explained by the selectivity of the cases approved by the board.^9^ ^-^ ^11^ The most prevalent errors identified here include tooth rotation, excessive buccolingual inclination, uneven marginal ridge, and occlusal relationship outside ideal intercuspation, all ranked in descending order of frequency. These results are close to the informal description provided by the ABO committee, although differing in the order specified.^1^

Teeth alignment errors (or rotation) were reported as the predominant factor contributing to suboptimal orthodontic finishing scores. These errors affected both anterior and posterior teeth, without distinction. Routine observations frequently noted that the lateral incisors and first and second molars were often among the most rotated teeth. Maxillary first molars finished in a Class II relationship may allow for some compensation in alignment, and must be contextualized. Except for this issue, the adoption of corrective measures is recommended to minimize these errors, such as rebonding brackets or making adjustments to the finishing archwires, paying special attention to more pronounced first-order bends.^1^ However, the control of premolar rotation poses challenges, especially in aligner-based orthodontic treatments, where it is a less predictable movement.^20^ Including hybrid mechanics or overcorrection in virtual planning can help correct premolar rotations, and thereby improve treatment efficiency.

The second most frequently identified error in orthodontic finishing was excessive buccolingual inclination, either labial or lingual, of posterior teeth. Ideally, there should be a minimal difference in height between the buccal and lingual cusps of upper and lower molars and upper premolars.^3^ This optimal buccolingual position promotes proper occlusal intercuspation and helps prevent balancing interferences. However, it is crucial to customize this positioning, based on individual skeletal morphology, and minor adjustments may be acceptable. The buccolingual inclination of molars can be influenced by pre-existing transverse and sagittal skeletal discrepancies prior to orthodontic treatment.^21^ In cases where orthodontic camouflage is the orthodontic approach, posterior torques can be preserved or even adjusted to compensate for more severe skeletal discrepancies. This may involve slight adjustments to the height of the buccal and lingual cusps, to achieve functional occlusion.

The third most frequently observed error was that of uneven marginal ridges. To address this issue, various approaches can be implemented, including rebonding brackets or incorporating second- and third-order bends in finishing archwires, depending on the etiology of the problem. Additionally, it is crucial to implement appropriate interarch mechanics to achieve optimal occlusion.^1^ Such strategies collectively contribute to improving the appearance and functional aspects of marginal ridges in the final occlusion, and minimize the absence of root parallelism.

There is a widespread view that second molars play a potentially critical role in orthodontic finishing. Despite the absence of specific data in this regard, the present study also corroborates that observation.^1^ ^,^ ^3^ Figure 1 (A, B, and C) shows common finishing errors involving second molars, including issues such as alignment, marginal ridge, and buccolingual inclination. The incorporation of second molars into orthodontic mechanics has indeed been neglected or improperly applied in several cases. Conversely, finishing errors in maxillary second molars have been attributed to bracket bonding errors, which result in excessive distal and lingual extrusion of the crown.^22^ When evaluating the normal position of the maxillary second molar in adolescents, it is important to consider that the crown may show mesial angulation and labial inclination, and that such position tends to change with age, thus resulting in a progressive uprighting of the tooth.^23^ Knowledge of these variations in the position of second molars with age is vital for leveling these teeth during treatment.^24^

The average total case complexity score in the current study, as measured by the DI, was 22.0, which indicates a high complexity level.^15^ ^,^ ^25^ This score was higher than that of an earlier study evaluating graduate orthodontic clinics, where the average DI score was 16.2.^25^ The high complexity score in the present study can be attributed to the minimum complexity score of 10, defined as the minimal score, and also the incorporation of three more cases with a minimum of 20 by the BBO, as well as the fact that orthodontic clinics involved in education often deal with more straightforward cases. However, as shown by this study and supported by others, the initial complexity of the case does not seem to be a reliable predictor of the quality of the outcome at the end of treatment.^13^ ^,^ ^25^ ^,^ ^26^ These findings suggest that case complexity, determined by the DI, may play an influential role in orthodontic planning but is not a determining factor in predicting the outcome. However, according to Kongboonvijit et al.^26^, regression analysis revealed that the severity of skeletal discrepancies, as determined by the Wits parameter, along with increased retroclination of the lower incisors and longer treatment duration, could negatively influence treatment outcomes. Other variables, such as the clinician’s skill and patient cooperation, may also influence the ultimate success of orthodontic treatments.

For educational purposes and to enhance orthodontic finishing with excellence, it would be advantageous to incorporate a checklist for orthodontic finishing, comprising three key recommendations. Firstly, include a comprehensive smile analysis, recognizing that assessing the occlusal outcome based solely on ABO standards may not reliably predict posttreatment smile attractiveness.^27^ The second recommendation refers to the assessment of functional occlusion. Given that the current examination primarily relies on static models, discrepancies between centric relation and maximum intercuspation (CR-MI) should be noted in the case description. Detecting and describing these functional requirements could reinforce orthodontic diagnosis and improve treatment outcomes, particularly regarding functional occlusion. The third recommendation emphasizes the importance of performing a comprehensive evaluation of occlusal and root parallelism using study models (cast, printed, or digital) and panoramic radiography, respectively, at the onset of the finalization phase, prior to appliance removal.^1^ ^,^ ^2^ In particular, multibracket fixed orthodontic appliances have been found to produce minimal distortions in digital models obtained via intraoral scanning.^28^ Thus, the CRE tool is invaluable in routine clinical practice and can facilitate both qualitative and quantitative assessments. 

Finally, improving orthodontic outcomes is a time-consuming process that can test patient resilience and satisfaction, particularly when inaccuracies arise during diagnosis, planning, and treatment management, or even a change of orthodontist in the course of treatment.^29^ An emerging consideration is how adherence to evidence-based practice could potentially limit the attainment of optimal orthodontic outcomes, especially when patient preferences play a significant role in clinical decision-making, notably in the final stage of the treatment. While this aspect remains relatively unexplored in the literature, it is recognized that patients’ perceptions are strongly influenced by aesthetic outcomes. It has been well established that patients’ perceptions before treatment may not always align with professional assessments,^30^ which gives rise to the following question: To what extent do occlusal details matter to patients and influence treatment outcome?

## LIMITATIONS

One methodological limitation that should be acknowledged in this study is its retrospective design, which did not allow for intra- and inter-examiner measurements applied for this research. Although data collection is objective and uses the ABO gauge, the evaluation may introduce inter-examiner variation, with some examiners potentially being more lenient than others. However, it is important to note that the BBO evaluation committee consists of eight experienced orthodontists who undergo rigorous calibration training and present good to excellent inter-examiner reliability, prior to the collection of the data. Furthermore, the evaluation process involved simultaneous assessment by pairs of examiners for each case presented. Such measures were implemented to minimize potential bias and ensure consistent and standardized evaluations.

Despite this limitation, the findings of the study provide a valuable insight into orthodontic finishing and highlight areas requiring attention and improvement to achieve optimal treatment outcomes.

## CONCLUSION

In summary, this study highlights the inevitability of orthodontic finishing errors, even in cases approved by the examination board. Additionally, it underscores the high sensitivity of the CRE method in detecting subtle changes. Common issues observed included tooth rotation, excessive buccolingual inclinations, and uneven marginal ridges, in that order. Starting with the premise that the diagnosis and treatment plan were adequate, optimizing occlusal refinement involves conducting a qualitative or quantitative assessment before removing the appliance. This can be achieved using physical casts or printed resin models, or digitalized intraoral images. Additionally, while case complexity, determined by DI, can impact orthodontic planning and pose challenges for clinicians, the study did not find it to be a determining factor in predicting treatment outcomes. 
